# Effect of Tightening Protocol on Reverse Torque and Plastic Deformation of Dynamic and Conventional Titanium Abutment Screws

**DOI:** 10.3390/bioengineering13101135

**Published:** 2026-09-28

**Authors:** Ricardo Batista, André Moreira, Rui B. Ruben, Maria Helena Figueiral, Nuno Alves, João Carlos Sampaio-Fernandes

**Affiliations:** 1 Faculty of Dental Medicine, University of Porto, Rua Dr. Manuel Pereira da Silva, 4200-393 Porto, Portugal; amoreira@fmd.up.pt (A.M.); mhsilva@fmd.up.pt (M.H.F.); jcsf@fmd.up.pt (J.C.S.-F.); 2 CDRSP, University of Leiria and Oeste, 2430-028 Leiria, Portugal; rui.ruben@ulo.pt (R.B.R.); nuno.alves@ulo.pt (N.A.); 3 INEGI—Institute of Science and Innovation in Mechanical and Industrial Engineering, Campus da FEUP, Rua Dr. Roberto Frias 400, 4200-465 Porto, Portugal

**Keywords:** dental implants, torque, dental prosthesis retention, mechanical stress, micro-computed tomography, abutment screw, preload, plastic deformation

## Abstract

Preload loss and plastic deformation of titanium abutment screws under repeated tightening are clinically significant yet incompletely characterized across systems with differing head geometries and surface treatments. Two ISO 14801-certified titanium screws were evaluated: hexalobular-head gold-plated (Gold-Tite^®^; 3i Zimmer Biomet, now ZIMVIE) and anodized star-shaped (Vega^®^; Klockner^®^). Six groups (*n* = 5 screws) varied by screw type, torque (20–40 N·cm), and cycle count (10 or 20). Reverse torque value (RTV) was recorded at every cycle and analyzed at the screw level; a linear mixed-effects model (screw as random effect) characterized how RTV evolved across cycles, with screw-level non-parametric tests for between-protocol contrasts. Micro-CT (19.9 μm; *n* = 3 per group) used Otsu segmentation. RTV was lower than insertion torque in every screw (30/30; 73–89% of applied torque). RTV declined significantly across cycles in the Vega/Klockner system (−0.17 N·cm per cycle, *p* = 0.006) but not in the ZFX/ZIMVIE system (*p* = 0.302), which showed greater between screw variability. Between protocol contrasts at *n* = 5 were null or underpowered. Micro-CT showed head localized change in both systems (mean cross-sectional area essentially unchanged); in the ZFX/ZIMVIE screws, this change appeared specifically under the over-use condition, whereas the Klockner screws showed comparable head change across all protocols. Preload loss is intrinsic to abutment screw tightening irrespective of design; RTV behaviour under repeated cycling is system-dependent, and abutment screws should be treated as limited-use components.

## 1. Introduction

Fixed implant-supported prostheses are retained by abutment screws that generate a clamping force, termed preload, at the implant–abutment interface through elastic deformation of the threaded joint [1]. Screw head geometries have evolved from single-groove designs to square, hexalobular, and star-shaped profiles, whereas shaft parameters such as thread pitch, diameter, and surface treatment vary considerably across systems [1]. Preload is the primary determinant of joint stability; when opposing forces, including occlusal imbalance or parafunctional habits, exceed the tightening force, screw loosening or complete loss of preload occurs [1,2,3,4,5,6,7]. Implant–abutment connection geometry modulates this relationship, and design strategies such as increased screw diameter, taller internal hexagons, and wider platform diameters have been introduced to improve resistance to loosening [1,8,9,10]. Retightening the screw five minutes and several weeks after initial placement is additionally recommended to compensate for the settling effect and limit preload loss [11,12].

Several operator and instrument-related factors influence torque delivery. The operator’s gender and body mass index affect the torque applied to the retaining screw [13,14], and the type of torque wrench introduces additional variability [13,15,16]. Preload is further modified by the use and type of lubricant, the physical properties of the contacting materials, and screw adjustment after initial tightening [17]. The screw itself constitutes an important mechanical variable: surface asperities increase friction and initial preload, whereas repeated removal and retightening progressively reduce these imperfections and, consequently, the preload retained at the interface [18,19].

The mechanical consequences of repeated screw tightening extend beyond preload variation. Excessive torque values and successive insertion cycles may promote screw loosening, plastic deformation, and fatigue fractures [18,20,21,22,23,24]. Using stereoscopic microscopy and micro-computed tomography (micro-CT), the authors showed that implant retaining screws undergo measurable plastic deformation after cyclic tightening. Because these deformation patterns differed by screw geometry and applied torque, the findings indicate that cumulative mechanical loading is a clinically relevant risk factor for screw integrity, independent of acute overload [22]. Consistent with this, Guzaitis et al. demonstrated that screws subjected to more than 10 insertion cycles showed significantly reduced reverse torque values and altered thread morphology, and recommended limiting the use to 10 cycles [24]. However, a more recent study found no statistically significant difference between tightening the abutment screw twice with or without a 10 min interval or only once [25], highlighting the need for further investigation into the influence of tightening protocols on screw behaviour.

When implant malposition or excessive angulation contraindicates conventional screw retention, angled screw channel abutments offer an alternative that allows angular correction of up to 30° without sacrificing retrievability [26]. Reported data on the mechanical behaviour of these abutments are conflicting. Mulla et al. found reverse torque values (RTVs), defined as the torque required to initiate reverse rotation of the abutment screw, comparable to those of straight-access abutments after cyclic loading [27], whereas Hu et al. reported significant differences in screw loosening between straight and angled configurations [28]. Across all connection geometries, RTV is the most widely used metric for quantifying preload retention and comparing the mechanical performance of different insertion protocols [25,27,28].

Despite the clinical relevance of tightening protocols on screw integrity, data comparing the combined effects of torque magnitude and cycle count on both preload retention and morphometric screw deformation across ISO-certified systems with differing head geometries and surface treatments remain limited. The primary objective of this study was to assess whether the screw type (dynamic or conventional) and tightening protocol independently influenced the RTV and produced measurable plastic deformation of the screw geometry, as characterized by micro-CT analysis. Four a priori null hypotheses were formulated (H_01_–H_04_);

H_01_. The RTV does not differ from the applied tightening torque value, regardless of screw type or insertion protocol.

H_02_. The RTV of identical screws does not vary with different tightening cycles or torque levels; that is, neither torque magnitude nor the number of insertion cycles affects the reverse torque of the same screw type.

H_03_. Different screws subjected to the same tightening protocol do not exhibit different RTVs; that is, screw type has no effect on reverse torque under equivalent mechanical loading.

H_04_. Repeated tightening does not produce measurable plastic deformation of the screw, regardless of torque or cycle count (assessed descriptively by micro-CT, given the small micro-CT sample).

The micro-CT morphometric outcomes addressed by H_04_ are, given the small micro-CT sample (n = 3 per group), reported descriptively rather than through formal significance testing.

## 2. Materials and Methods

This study was conducted entirely in vitro, using standardized mechanical components. No human participants or animal subjects were involved; therefore, demographic variables (age, gender, race) and sex/gender reporting requirements were not applicable. Operator-related variability, including gender-associated differences in manual torque application, was eliminated by the exclusive use of a calibrated electronic torque wrench throughout all experimental procedures.

### 2.1. Specimen Selection and Experimental Groups

Two types of commercially available ISO 14801-certified [29] titanium abutment screws were evaluated: (i) hexalobular-head gold-plated titanium screws (Gold-Tite®; 3i Zimmer Biomet, Warsaw, IN, USA) and (ii) anodized titanium screws with a star-shaped head (Vega®; Klockner®, Sant Boi de Llobregat, Spain). Fifteen screws of each type/brand were used. Six experimental groups were defined according to screw type, applied torque, and number of tightening cycles, with five screws per group (Table 1).

For the ZFX^®^/ZIMVIE^®^ system, three protocols were tested: the manufacturer-recommended protocol (Group D1:10 cycles at 20 N·cm), an overuse condition (Group D2:20 cycles at 20 N·cm), and an over-torque condition (Group D3:10 cycles at 30 N·cm). For the Klockner^®^ system, three conditions bracketing the manufacturer-recommended torque of 30 N·cm were applied: the recommended protocol (Group V1:10 cycles at 30 N·cm), an overuse condition (Group V2:20 cycles at 30 N·cm), and an over-torque condition (Group V3:10 cycles at 40 N·cm). This factorial design was intended to dissociate the independent contributions of torque magnitude and cycle count to the screw deformation within each system (Table 2). All materials used in each group are listed in Table 2.

Throughout this article, the ZFX/ZIMVIE (Gold-Tite) hexalobular screw is designated the dynamic screw and the Vega/Klockner star-shaped screw the conventional-connection screw, consistent with the terminology used in the title.

The reverse-torque and micro-CT data reported here were obtained within a larger doctoral experimental programme that used a shared component sample. A separate subset of that programme, addressing screwdriver efficacy across repeated tightening/untightening cycles, has been published previously [30]; the present study analyses a distinct set of screw groups (ZFX/ZIMVIE and Vega/Klockner abutment screws) with different outcomes (reverse torque and micro-CT deformation) and does not overlap in results with that report.

### 2.2. Tightening Protocol and Laboratory Procedure

All experiments were conducted under standardized laboratory conditions by a single experienced operator. The receiving components were manufacturer dummy implants (Klockner and ZFX/ZIMVIE) of the same titanium alloy as the corresponding clinical implants, confirmed against the manufacturers’ technical specifications. For each group, five abutment screws were tested, each in its own new dummy implant (30 implants in total); within a group a single abutment was used across the five screws while the receiving implant was replaced for every screw, which prevents progressive modification of the implant internal threads from confounding RTV across the screw sequence [30]. Dental implants were embedded in self-curing acrylic resin at 90° to ensure reproducible axial load transfer. Each screw was tightened according to the group-specific protocol (10 or 20 consecutive cycles at 20, 30, or 40 N·cm) using a calibrated cordless electronic torque wrench (NSK iSD900). Repeat tightening at the same torque was applied 10 min after the final cycle. The reverse torque value (RTV) was recorded with a calibrated digital torque screwdriver at each loosening event across the cycle sequence, providing a within-screw series of measurements per screw (Figure 1).

### 2.3. Reverse Torque Value Measurement and Statistical Analysis

The experimental unit was the individual screw (*n* = 5 per group). Because each screw produced a sequence of RTV readings across cycles, these repeated measurements were not treated as independent. Normality was assessed per group (Shapiro–Wilk, Kolmogorov–Smirnov), and non-parametric tests were used where appropriate (α = 0.05). The primary analysis was a linear mixed-effects model, fitted separately for each system, which relates RTV to the cycle number while accounting for the fact that the repeated readings belong to the same five screws; this uses all measurements without treating them as independent observations. Secondary between-protocol comparisons used the Mann–Whitney U test on each screw’s mean RTV (five screws versus five). The mean RTV per screw was chosen in advance as the main summary because it represents each screw’s typical behaviour across the whole protocol rather than a single moment; each screw’s final-cycle RTV was analyzed the same way as a sensitivity check. The comparison of RTV against the applied insertion torque (H_01_) used the Wilcoxon signed-rank test at the screw level.

### 2.4. Micro-Computed Tomography Acquisition

Morphometric analysis of screw geometry was performed by micro-computed tomography (micro-CT) at MicronSense (Leiria, Portugal) using a GE Phoenix v|tome|x m scanner (GE Sensing & Inspection Technologies GmbH, Wunstorf, Germany) with an isotropic voxel resolution of 19.9 μm. For each implant system, two unused screws served as unloaded reference specimens. Three screws were randomly selected from each experimental group for scanning. Each specimen was reconstructed across a series of transverse cross-sections, yielding 45–48 slices per specimen for the Klockner^®^ system and 30–34 slices for the ZFX^®^/ZIMVIE^®^ system, representing the full axial extent from the screw head to the thread terminus. Within each reconstruction, the two most coronal sections corresponded to the screwdriver engagement region of the screw head, distinct from the functional tightening zone engaged by the abutment–implant interface. These two sections were excluded a priori from all morphometric and statistical analyses described below, for both the reference specimens and the experimental groups, so that the reported outcomes reflect plastic strain within the functional tightening zone rather than the non-functional driver-contact region.

### 2.5. Image Segmentation and Cross-Sectional Area Measurement

Screw geometry was quantified from micro-CT reconstructions using a custom Python pipeline (Python 3.11) based on Otsu’s global thresholding method [22], as previously described. For each scan, the device generated pairs of 8-bit grayscale bitmap images (256 intensity levels, 0–255), one per transverse slice. The optimal intensity threshold k* is determined by maximizing the between-class variance σ2ʙ:

σ^2^ʙ(k*) = max_0_ ≤ k≤ _255_ σ^2^ʙ(k), where σ^2^ʙ(k) = (mɢ·P_1_ − m)^2^/[P_1_(1 − P_1_)]

where mɢ is the mean intensity of all pixels, m is the cumulative mean intensity up to threshold k, and P_1_ is the normalized cumulative histogram up to intensity level k. The number of pixels classified as belonging to the screw cross-section was determined for each slice and converted to the physical area (mm^2^) from the known pixel pitch (19.9 μm). The first slices in each reconstruction corresponded to the screw head, and the terminal slices corresponded to the thread region (Figure 2).

### 2.6. Morphometric Outcomes

From the segmented slice stack of each screw, the minimum, maximum, and mean cross-sectional areas (mm^2^) and the total reconstructed volume (mm^3^) were derived. Deformation was quantified as the relative change in cross-sectional area of a tightened screw with respect to the mean profile of the two reference screws, computed section by section to remove the effect of the conical shaft geometry:

Deformation (%) = [(Ascrew − Āreference)/Āreference] × 100

where Ascrew is the cross-sectional area of the loaded screw at a given section, and Āreference is the mean cross-sectional area of the corresponding section in the undeformed reference screw.

### 2.7. Statistical Analysis of Morphometric Data

Micro-CT outcomes were analyzed separately for each implant system (IBM SPSS Statistics v27; IBM Corp., Armonk, NY, USA), on the dataset from which the two screwdriver-engagement sections had been excluded (Section 2.4). Given the small sample (three specimens per condition), the morphometric outcomes are reported descriptively rather than through formal significance testing: for each condition, we report the mean value, the relative change from the unloaded reference, and standardized effect sizes (Cohen’s d) as a measure of how large the differences are. The maximum cross-sectional area at the screw head was the primary descriptor in both systems, because change was concentrated at the head; the mean cross-sectional area and the reconstructed volume were secondary descriptors. Conditions compared were, for ZFX/ZIMVIE, the recommended (10 × 20 N·cm), over-torque (10 × 30 N·cm), and over-use (20 × 20 N·cm) protocols, and, for Vega/Klockner, the recommended (10 × 30 N·cm), over-use (20 × 30 N·cm), and over-torque (10 × 40 N·cm) protocols.

## 3. Results

### 3.1. Reverse Torque Values

The experimental unit for all reverse-torque analyses is the individual screw (*n* = 5 per group). Descriptive statistics per group are given in Table 3. Because each screw was measured at every cycle, the primary analysis modelled the full within-screw sequence with a mixed-effects model, and between-protocol contrasts were performed at the screw level.

RTV relative to insertion torque (H_01_)

Every screw returned RTV below its applied insertion torque (30/30; median 87%, group medians 80–90%). Because all five screws in each group fell on the same side of the applied torque, the screw-level Wilcoxon test reaches the value of *p* = 0.062; the result is reported as a uniform, directionally consistent reduction rather than a significance test.

Evolution of RTV across cycles (primary outcome)

RTV declined significantly across cycles in the Vega/Klockner system (slope −0.17 N·cm/cycle; 95% CI −0.29 to −0.05; *p* = 0.006), but not in the ZFX/ZIMVIE system (−0.11; 95% CI −0.31 to +0.10; *p* = 0.302), which showed markedly greater between-screw variability. The systems differed in the stability of RTV under repeated cycling (Figure 3).

Between-protocol contrasts (secondary, exploratory)

Doubling the cycles at 30 N·cm did not change RTV in the Vega/Klockner system (10 × 30 vs. 20 × 30; *p* = 0.84), and the two systems did not differ under the matched 10 × 30 N·cm protocol (*p* = 1.00). Two comparisons did reach significance on the mean RTV (Vega/Klockner 10 × 30 vs. 10 × 40, and ZFX/ZIMVIE 10 × 20 vs. 20 × 20; both *p* = 0.008); however, each of these rested on the five screws of one group being entirely separated from the five of the other, which is the most fragile way a small sample can reach significance, and neither result held when the screws were summarized by their final-cycle value instead of their mean (*p* = 0.29). These two comparisons are therefore reported as exploratory.

### 3.2. Micro-CT Morphometric Analysis: Vega/Klockner System

For each specimen, the cross-sectional area was defined as the region enclosed by the outer contour of the screw section (the hexagonal connection profile shown in Figure 2), measured perpendicular to its long axis on the micro-CT reconstructions. This area was quantified as the number of pixels contained within that contour, providing a direct measure of the amount of material within a given transverse plane, so that any plastic deformation of the screw is reflected as a measurable change in the enclosed pixel area.

Eleven Vega/Klockner screws were analyzed (two references, nine experimental, *n* = 3 per group) (Figure 4). The pooled within-group coefficient of variation in the mean cross-sectional area was 0.98%, consistent with specimen homogeneity within ISO 14801 tolerances. Relative to the unloaded reference, mean cross-sectional area increased in all three loaded groups (Table 4): +3.6% at 10 × 30 N·cm, +4.6% at 10 × 40 N·cm, and +1.7% at 20 × 30 N·cm. At a fixed 10 cycles, the change was larger at higher torque (10 × 30 vs. 10 × 40), whereas doubling the cycles at 30 N·cm (10 × 30 vs. 20 × 30) was associated with a smaller mean change rather than a larger one. Given *n* = 3 per group, these differences are reported descriptively, with effect sizes, rather than as hypothesis tests, and the non-monotonic cycle result should be interpreted cautiously as it may reflect between-specimen variability at this sample size.

Standardized effect sizes (Cohen’s d) for the mean cross-sectional area were large for the widest torque comparisons (1.9 for 20 vs. 30 N·cm and 2.9 for 20 vs. 40 N·cm) and moderate for the closest one (0.9 for 30 vs. 40 N·cm). With only three specimens per group, however, these comparisons are underpowered and are therefore treated as exploratory; a larger sample (about 22 screws per group) would be needed to test adjacent-torque differences reliably.

### 3.3. Micro-CT Morphometric Analysis: ZFX/ZIMVIE System

Eleven ZFX/ZIMVIE screws were analyzed (two references, nine experimental, *n* = 3 per protocol) (Figure 5). Across all three protocols, the mean cross-sectional area was essentially unchanged relative to the reference (−0.4% to −0.8%; small negative values consistent with measurement noise rather than true loss of material) (Figure 6). Change was instead confined to the maximum area at the screw-head region: the recommended (10 × 20) and over-torque (10 × 30) conditions showed negligible head change (+0.3% and +0.1%; Table 5), whereas the overuse condition (20 × 20) showed a marked head-region change (maximum area +8.2%; range across specimens +4.3% to +13.1%; Table 6). Given *n* = 3 per group, these are reported descriptively with effect sizes rather than as hypothesis tests; the pattern indicates that, in this system, change was concentrated at the head under the overuse condition rather than following torque magnitude.

Pairwise effect sizes for maximum cross-sectional area were trivial for 10 × 20 vs. 10 × 30 (Cohen’s d = 0.08) and very large for comparisons involving 20 × 20 (d = 2.38 and 2.46). An a priori estimate indicated that *n* = 5 specimens per condition would be needed to detect the recommended-versus-overuse contrast at 80% power, which the present *n* = 3 does not reach.

### 3.4. Summary of Morphometric Findings

Across both implant systems, screwdriver-engagement sections were excluded from all morphometric analyse, so that reported change reflects the functional tightening zone. In both systems, the mean cross-sectional area remained essentially unchanged, while change was concentrated at the maximum cross-sectional area (the head region); deformation was thus localized rather than distributed along the shaft in both screws. The two systems differed, however, in how deformation related to the loading protocol. In the ZFX/ZIMVIE system, change at the head was clearly associated with the number of tightening cycles: only the over-use condition (20 × 20 N·cm) produced a marked increase (+8.4%), whereas the recommended and over-torque conditions did not (−0.7% and −1.0%). In the Klockner system, all protocols, including the recommended one, produced head-region changes of comparable magnitude (+3.6% to +6.8%), with no protocol clearly separating from the others; at *n* = 3 per group, this precludes attributing the Klockner deformation to any single factor. All values are descriptive and hypothesis-generating given the sample size.

## 4. Discussion

The universality of preload loss across all experimental conditions, irrespective of screw type, head geometry, or surface treatment, confirms that this phenomenon is an intrinsic mechanical property of the threaded-fastener system rather than an artifact of any particular component design. This inference is consistent with the settling effect described by Winkler et al. [3], in which the microplastic deformation of surface asperities at the thread and abutment–implant interfaces dissipates a fraction of the applied torque, rendering the retrieval torque systematically lower than the insertion torque. The present ratios are comparable to those reported by Alfaifi et al. [9] and are consistent with the tribological observations of Batista et al. [22], who attributed the lower frictional resistance of gold-plated screws to reduced surface roughness. The practical consequence is that insertion torque values should be understood as lower bounds of the intended preload rather than precise targets for the clamping force retained at the interface during function.

The benefit of repeated tightening cycles on preload retention was confined to one screw system, which has a mechanistic implication that extends beyond the specific components tested: the settling advantage is surface- and geometry-dependent and cannot be generalized across implant platforms. The absence of a cycle-count benefit in the anodized star-shaped screws is most plausibly attributable to the lower initial roughness and greater chemical stability of the anodized titanium oxide layer, which may limit the scope for further asperity smoothing under repeated driver engagement. This is a speculative interpretation, since direct tribometric surface measurements (e.g., profilometry or scanning electron microscopy) were not performed; however, it is supported by established literature on anodized titanium tribology [17,19]. Separately, the micro-CT data indicate that repeated tightening produces measurable, head-localized change in both systems, with the ZFX/ZIMVIE screws showing this change specifically under the over-use (repeated-cycle) condition. The broader implication that tightening protocol influences both preload retention and screw geometry reinforces the clinical priority of protocol standardization, as previously argued by Alnasser et al. [25].

Micro-CT showed that, in both systems, repeated tightening left the mean cross-sectional area essentially unchanged while producing localized change at the head region; the two screws therefore shared a common, head-localized pattern rather than two distinct deformation modes. They differed in how this change related to the protocol: in the ZFX/ZIMVIE screws the head change was clearly associated with the number of tightening cycles (marked only under the over-use condition), whereas in the Klockner screws all protocols, including the recommended one, produced head change of comparable magnitude, so that no single factor could be isolated at this sample size. These observations extend the qualitative findings of Batista et al. [22], but should be read as descriptive given *n* = 3 per group. The reverse-torque data showed a separate system-dependence: RTV declined progressively across cycles in the Vega/Klockner screws yet remained without a detectable systematic trend, though more variable, in the ZFX/ZIMVIE screws. Characterizing RTV as a trajectory across cycles, rather than a single endpoint, is what the present design was intended to capture.

With only five screws per group, the between-protocol comparisons were either non-significant or, when they did reach significance, rested on a perfect but fragile separation between the two small groups, a result that did not hold when the screws were summarized in a slightly different way. The study is therefore not powered to define protocol-specific reverse-torque thresholds. This qualifies, but does not overturn, the clinical observation that repeated tightening measurably affects both the retrievable preload and the screw geometry.

The observation that plastic deformation occurred in one system even within the clinically recommended torque range, and in the other under a cycle-count condition readily reached during provisional and try-in phases, supports treating titanium abutment screws as limited-use components. On the present evidence we do not propose specific numeric replacement thresholds, which the sample size does not support; this is nonetheless consistent with the guidance of Guzaitis et al. [24] and Batista et al. [22] and is particularly relevant in rehabilitation workflows, where repeated screw removal is inherent [31]. A complementary perspective on this clinical risk was provided by Batista et al. [30], whose parallel protocols showed a progressive RTV decline with reuse of the same screwdriver and implant. These findings support viewing the implant–abutment screw assembly as a set of interacting wear surfaces whose cumulative history governs preload retention.

Gender is a recognized source of variability in clinician-generated torque on abutment screws, with both gender and body mass index shown to influence manually applied force [13,14]. The exclusive use of a calibrated electronic torque wrench in the present study a decision shared with Batista et al. [30] removed gender-related operator variability, ensuring that observed differences in RTV and deformation reflect component and protocol properties rather than operator characteristics. Consequently, the findings cannot be directly extrapolated to clinical settings where manual screwdrivers remain in use, in which gender-related differences in delivered torque may systematically modify preload retention and screw deformation. Future investigations should incorporate multi-operator designs stratified by gender and instrumentation type to address this.

This study has several limitations. The receiving components were dummy implants of the same titanium alloy as the clinical implants, differing only in the external osseointegrative surface treatment; because all measurements concern the internal screw–implant interface and the abutment screw itself, this external difference is not expected to affect the tribological or deformation outcomes, although absolute RTVs should be read as representative of the internal connection rather than transferred uncritically to every clinical implant surface. A new implant was used for each screw (30 in total); this design directly addresses a limitation we identified in our earlier stereoscopic-microscopy and micro-CT study [22], in which the same implant was reused within each group and a new implant per screw was recommended for future work it controls the corresponding wear confound, since RTV declines when the same implant is reused but not when a new implant is used per screw [30]. A residual confound remains, as a single abutment was shared across the five screws within a group, so abutment interface wear cannot be fully excluded, although its expected magnitude is smaller than implant-thread wear. The absence of functional occlusal loading means the data capture only the tribological and geometric consequences of tightening, not fatigue behaviour. Finally, the sample of five screws per group supports the within-screw trajectory analysis but limits power for between-group contrasts, which are therefore reported as exploratory; this sample size is nonetheless consistent with comparable invitro studies of abutment screw deformation and reverse torque, including our own previous work [22,30] and earlier reports [18], in which five specimens per group were used.

Future studies should incorporate functional cyclic loading, dedicated implant–abutment assemblies per specimen, direct tribometry surface characterization before and after tightening, larger screw samples, and multi-operator designs stratified by gender and instrumentation type. Extending the morphometric micro-CT approach to internal conical and Morse taper connections would establish whether the head-localized deformation observed here reflects broader biomechanical principles or is specific to external-connection geometries. Given that measurable plastic deformation was observed under repeated tightening, further work could also examine how repeated-tightening behaviour relates to current certification testing, which is not designed around this specific loading history.

## 5. Conclusions

Within the limitations of this in vitro study:

(1) Reverse torque was uniformly lower than insertion torque (30/30 screws; median 87%). Final tightening torque should be regarded as an upper bound on retrievable preload. Calibrated electronic torque delivery is preferred to minimize variability.

(2) RTV evolves across repeated cycles in a system-dependent way: a significant progressive decline in the anodized star-shaped Vega/Klockner screw, versus no significant systematic decline but greater variability in the gold-plated hexalobular ZFX/ZIMVIE screw.

(3) Between-protocol differences were not robust at *n* = 5; the study is not powered for protocol-specific RTV thresholds, and system-specific replacement thresholds are not claimed.

(4) Repeated tightening produced measurable, head-localized plastic deformation in both systems (micro-CT), with the mean cross-sectional area essentially unchanged. In the ZFX/ZIMVIE screws, this change was associated with the number of tightening cycles (over-use condition); in the Klockner screws, all protocols produced comparable head change, precluding attribution to a single factor at *n* = 3. These observations motivate further study of how repeated-tightening behaviour relates to current certification testing, without drawing conclusions here about any specific standard.

(5) Abutment screws behave as limited-use components; screws subjected to repeated removal (provisional and try-in phases) should be treated as consumable. System-specific quantitative thresholds await adequately powered confirmation.

## Figures and Tables

**Figure 1:**
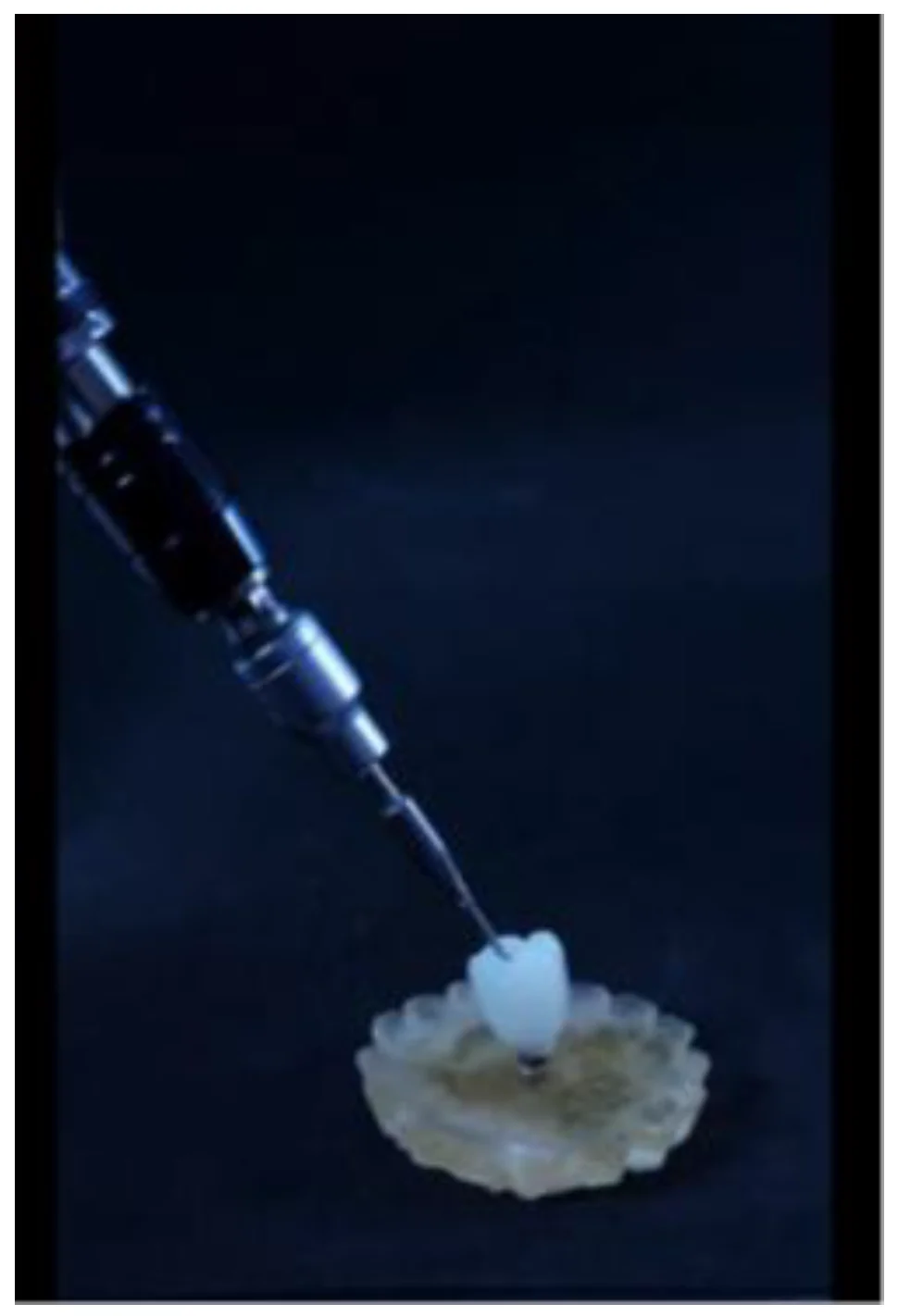
Experimental setting: a dental implant fixed in self-cured acrylic resin was untightened from a dynamic internal connection abutment (4 mm platform; ZFX^®^/ZIMVIE^®^) with a PMMA crown at 20° angulation using a calibrated digital torque screwdriver.

**Figure 2:**
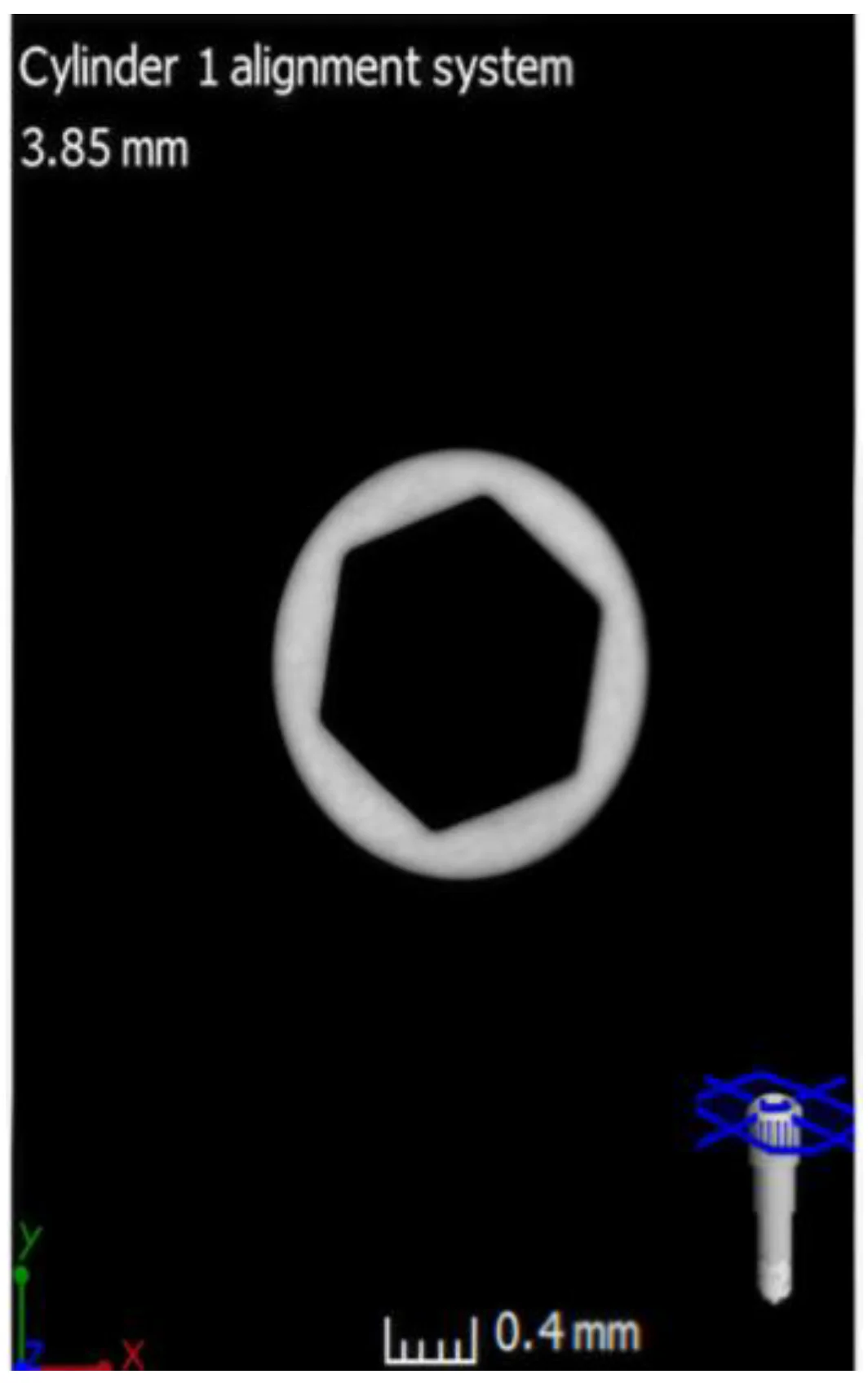
Representative micro-CT image showing a transverse cross-section at the screw-head level, illustrating the binary segmentation used for the cross-sectional area measurement. Scale bar = 0.4 mm.

**Figure 3:**
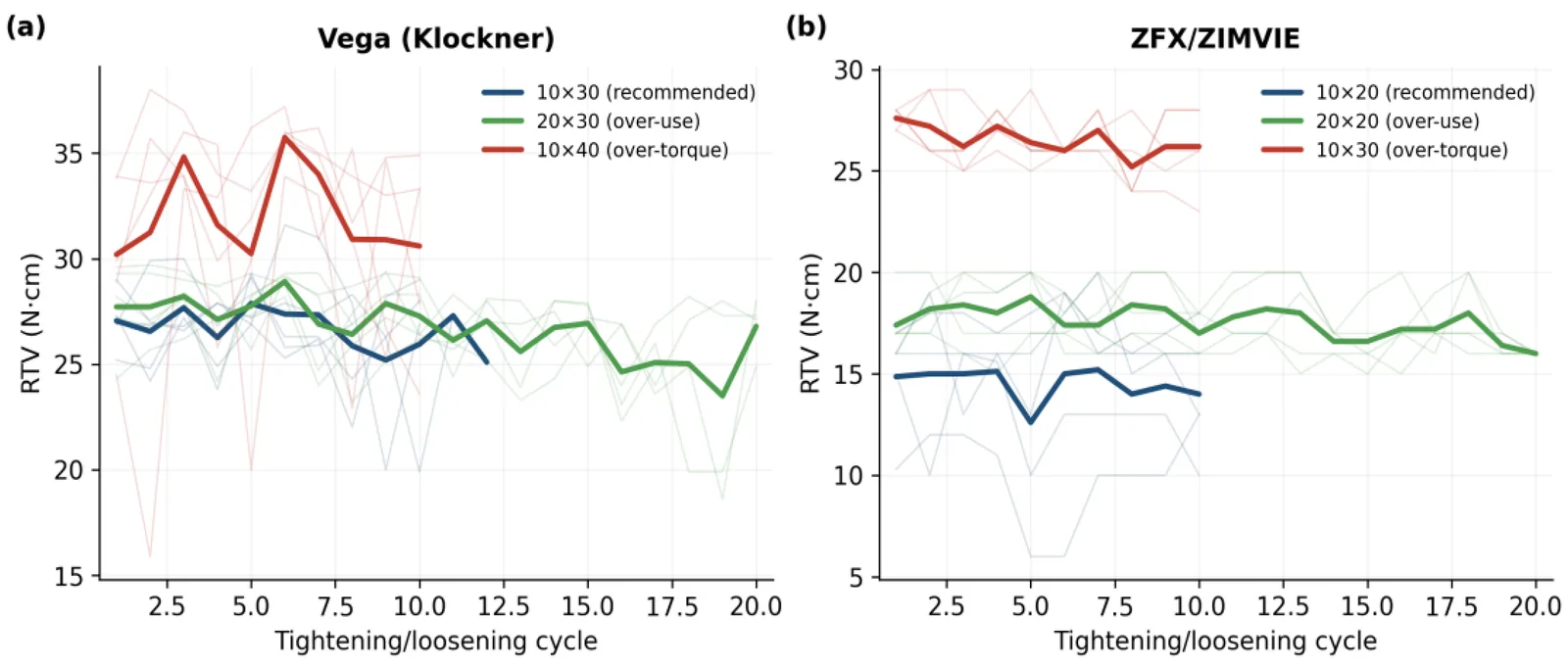
Reverse torque value (RTV) across successive tightening/loosening cycles, shown per screw for each system: (**a**) Vega/Klockner; (**b**) ZFX/ZIMVIE. Within each panel, the three protocols are shown in different colours (recommended, over-use, over-torque); thin lines are individual screws (*n* = 5 per group) and bold lines are the per-protocol mean per cycle. In a linear mixed-effects model (screw as random effect), RTV declined significantly across cycles in the Vega/Klockner system (slope −0.17 N·cm per cycle; 95% CI −0.29 to −0.05; *p* = 0.006) but showed no significant systematic trend in the ZFX/ZIMVIE system (slope −0.11 N·cm per cycle; 95% CI −0.31 to +0.10; *p* = 0.302). Note the different vertical scales between panels.

**Figure 4:**
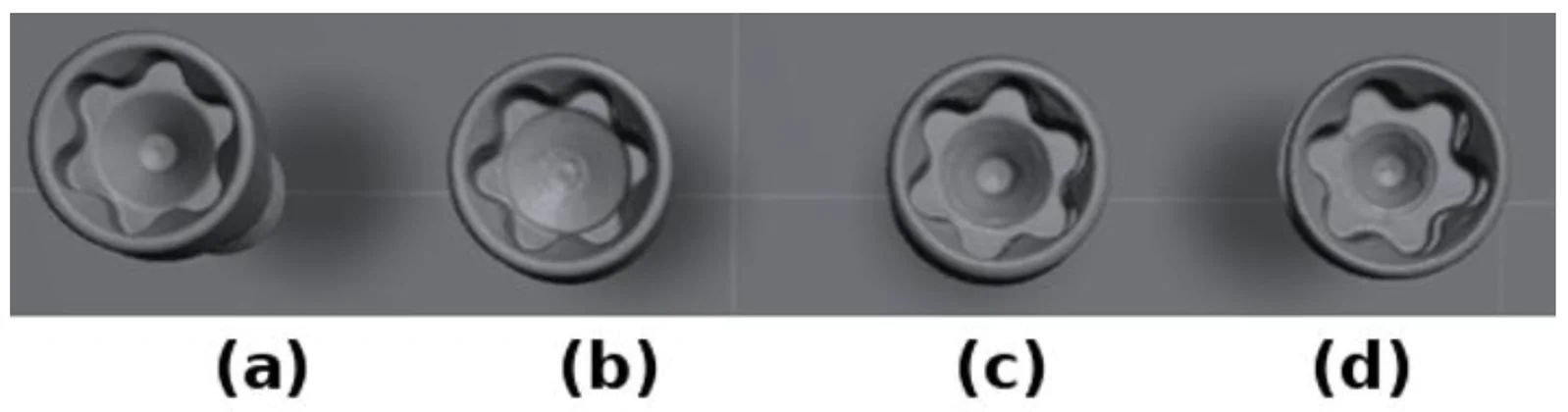
Three-dimensional micro-CT reconstructions of Klockner^®^ (Vega) abutment screws: (**a**) unloaded reference; (**b**) 20 cycles at 30 N·cm; (**c**) 10 cycles at 30 N·cm; (**d**) 10 cycles at 40 N·cm.

**Figure 5:**
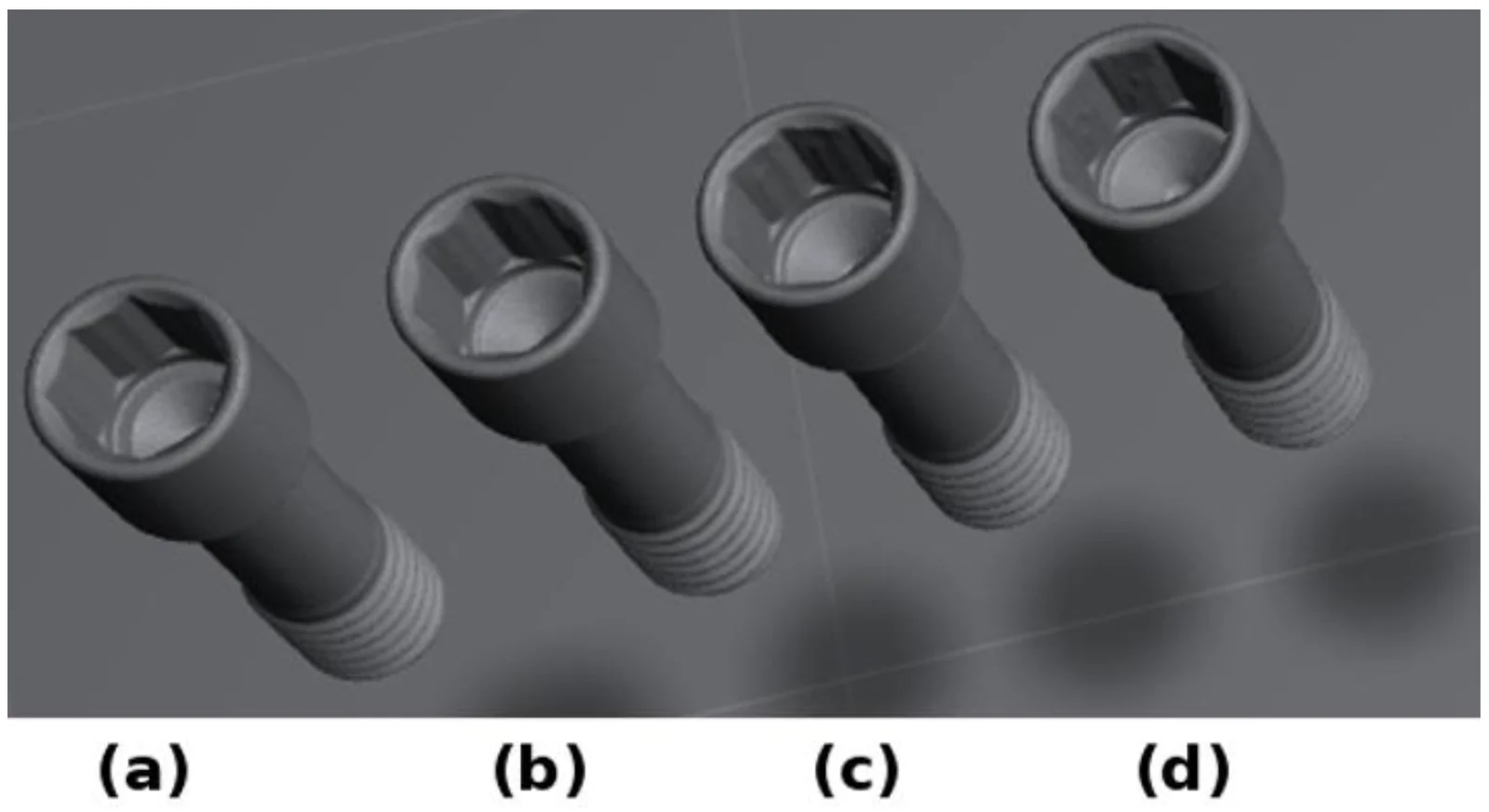
Three-dimensional micro-CT reconstructions of ZFX^®^/ZIMVIE^®^ abutment screws: (**a**) unloaded reference; (**b**) 10 cycles at 20 N·cm (10 × 20); (**c**) 10 cycles at 30 N·cm (10 × 30); (**d**) 20 cycles at 20 N·cm (20 × 20).

**Figure 6:**
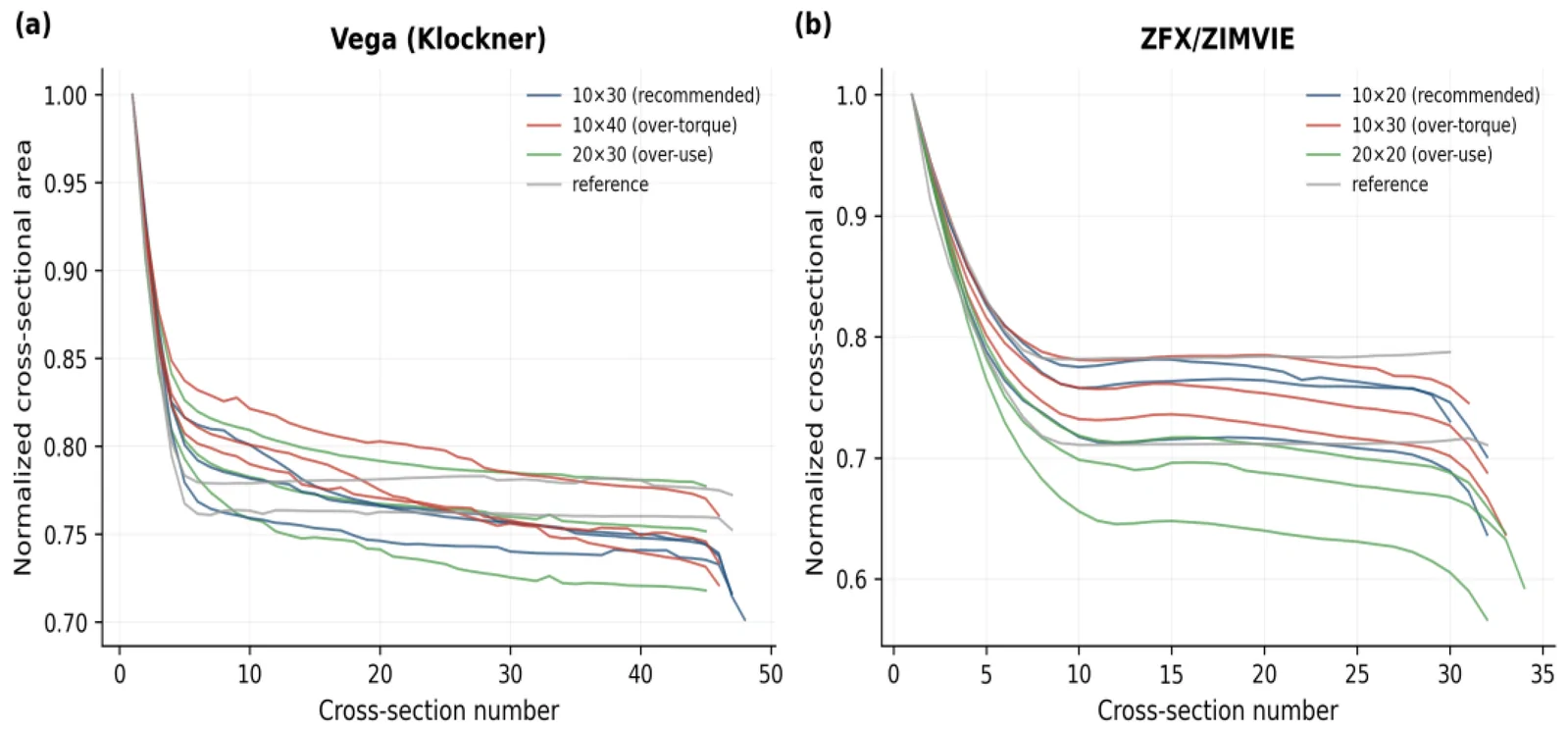
Normalized cross-sectional area profiles along the screw long axis, obtained from micro-CT reconstructions, for each individual specimen: (**a**) Klockner^®^ system; (**b**) ZFX^®^/ZIMVIE^®^ system. Each curve represents one screw, with the cross-sectional area normalized to its first included section. The two topmost sections (screwdriver-engagement zone) were excluded from every specimen. Reference (new, unloaded) screws are labelled “new-1” and “new-2”; experimental screws are labelled by tightening protocol (cycles × torque) and specimen number. Note the different vertical scales between panels, reflecting the proportionally larger head of the ZFX^®^/ZIMVIE^®^ screws.

**Table 1:** Experimental groups: screw type, tightening torque, number of cycles, and specimen count.

Group	Screw Type	Torque (N·cm)	Cycles (*n*)	Screws (*n*)	RTV Measurements (*n*)
D1	Hexalobular gold-plated (Gold-Tite^®^; ZFX^®^/ZIMVIE^®^)	20	10	5	50
D2	Hexalobular gold-plated (Gold-Tite^®^; ZFX^®^/ZIMVIE^®^)	20	20	5	100
D3	Hexalobular gold-plated (Gold-Tite^®^; ZFX^®^/ZIMVIE^®^)	30	10	5	50
V1	Anodized star-shaped (Vega^®^; Klockner^®^)	30	10	5	50
V2	Anodized star-shaped (Vega^®^; Klockner^®^)	30	20	5	100
V3	Anodized star-shaped (Vega^®^; Klockner^®^)	40	10	5	50

Groups D1–D3: ZFX^®^/ZIMVIE^®^ hexalobular gold-plated screws. Groups V1–V3: Klockner^®^ anodized star-shaped screws. Manufacturer-recommended torques: 20 N·cm (ZFX^®^/ZIMVIE^®^); 30 N·cm (Klockner^®^).

**Table 2:** Components used in each experimental group.

Group D—Hexalobular Gold-Plated (Gold-Tite^®^; ZFX^®^/ZIMVIE^®^; 3i Zimmer Biomet, Warsaw, IN, USA)	Group V—Anodized Star-Shaped (Vega^®^; Klockner^®^, Sant Boi de Llobregat, Spain)
15 dummy internal-connection implants (one new dummy implant per screw; same titanium alloy as the clinical implant), 4 mm platform, 11.5 mm length (based on Certain; 3i Zimmer Biomet, Warsaw, IN, USA)	15 dummy internal-connection implants (one new dummy implant per screw; same titanium alloy as the clinical implant), 4 mm platform, 12 mm length (based on Vega^®^ ref. 194112; Klockner^®^)
1 dynamic internal-connection abutment with PMMA crown, 4 mm platform, 20° angulation (ZFX11ZB-CE41ASZ03E; ZFX GmbH/ZIMVIE, Dachau, Germany)	1 internal-connection abutment, 4 mm platform (Vega^®^ ref. 840105; Klockner^®^)
15 hexalobular gold-coated titanium retaining screws (Gold-Tite^®^ ref. ZFX09-ZB-CE-HLGTN; 3i Zimmer Biomet)	15 anodized titanium retaining screws (Vega^®^; Klockner^®^)
15 hexalobular screwdrivers (ref. ZFX02HLD28; 3i Zimmer Biomet)	15 star-shaped torque wrench adapters (ref. 100811L; Klockner^®^)
Cordless electronic torque wrench (NSK iSD900; NSK Nakanishi Inc., Kanuma, Japan); digital torque screwdriver (Tapha Tools SD/SP; Tapha Co., Shenzhen, China)	Cordless electronic torque wrench (NSK iSD900; NSK Nakanishi Inc., Kanuma, Japan); digital torque screwdriver (Tapha Tools SD/SP; Tapha Co., Shenzhen, China)

A new dummy implant was used for each screw (15 per group, 30 in total); dummy implants share the same titanium alloy as the clinical implants and differ only in the external osseointegrative surface treatment, which does not involve the measured internal screw–implant interface.

**Table 3:** Reverse torque value (N·cm) per group—per-screw mean, *n* = 5.

Group	*N*	Median	IQR (Q1–Q3)	Min–Max
ZFX/ZIMVIE 10 × 20 N	5	16.40	12.86–16.50	10.03–16.80
ZFX/ZIMVIE 20 × 20 N	5	17.55	17.05–17.70	16.95–18.55
ZFX/ZIMVIE 10 × 30 N	5	26.80	26.70–26.80	25.20–27.10
Vega/Klockner 10 × 30 N	5	26.65	26.19–27.28	26.12–27.39
Vega/Klockner 20 × 30 N	5	26.56	26.46–27.23	25.82–27.29
Vega/Klockner 10 × 40 N	5	32.77	30.67–33.44	29.64–33.61

ZFX/ZIMVIE = Gold-Tite (3i Zimmer Biomet/ZIMVIE); Vega = Klockner.

**Table 4:** Descriptive micro-CT morphometric outcomes for the Klockner^®^ (Vega) system, group means (*n* = 3 per group).

Protocol (Cycles × N·cm)	Δ Mean Area (%)	Δ Volume (%)
Reference (unloaded, *n* = 2)	—	—
10 × 30—recommended	+3.64	+4.14
20 × 30—over-use	+1.66	−2.56
10 × 40—over-torque	+4.55	+2.51

Δ: relative change from the mean of the two unloaded reference screws. Values are group means of three replicates. The apparent volume decrease in the 20 × 30 group reflects a lower number of reconstructed cross-sections (45 vs. 47) rather than true volume loss; mean cross-sectional area, normalized per section, is the reference deformation metric. Given *n* = 3 per group, values are descriptive; see Section 3.2.

**Table 5:** Descriptive micro-CT morphometric outcomes for the ZFX^®^/ZIMVIE^®^ system, group means (*n* = 3 per group).

Protocol	Δ Max Area—Head (%)	Δ Mean Area (%)	Δ Volume (%)
Reference (unloaded, *n* = 2)	—	—	—
10 × 20—recommended	−0.74	−0.73	+0.62
10 × 30—over-torque	−1.04	−0.75	+2.62
20 × 20—overuse	+8.42	−0.44	+6.23

Δ: relative change from the mean of the two unloaded reference screws. Maximum area at the head is the pre-specified primary descriptor because deformation was localized to the head and superior thread region. Mean-area changes are near zero (essentially unchanged). The dissociation between Δ Max area (+8.42%) and Δ Mean area (−0.44%) in the overuse group confirms localized rather than diffuse deformation. Given *n* = 3 per group, values are descriptive; see Section 3.3.

**Table 6:** Per-specimen maximum cross-sectional area, Klockner^®^ (V) and ZFX^®^/ZIMVIE^®^ (D) systems.

Specimen	Condition	Max Area (mm^2^)	Δ vs. Ref (%)
20x30_1 (V)	30 N·cm—over-use	2.213	+1.1
20x30_2 (V)	30 N·cm—over-use	2.317	+5.9
20x30_3 (V)	30 N·cm—over-use	2.272	+3.8
10x30_1 (V)	30 N·cm—recommended	2.314	+5.7
10x30_4 (V)	30 N·cm—recommended	2.347	+7.2
10x30_5 (V)	30 N·cm—recommended	2.350	+7.4
10x40_2 (V)	40 N·cm—over-torque	2.368	+8.2
10x40_3 (V)	40 N·cm—over-torque	2.299	+5.0
10x40_4 (V)	40 N·cm—over-torque	2.276	+4.0
10x20_1 (D)	10 × 20—recommended	2.427	+2.6
10x20_2 (D)	10 × 20—recommended	2.353	−0.5
10x20_5 (D)	10 × 20—recommended	2.338	−1.2
10x30_1 (D)	10 × 30—over-torque	2.358	−0.3
10x30_3 (D)	10 × 30—over-torque	2.393	+1.2
10x30_4 (D)	10 × 30—over-torque	2.354	−0.5
20x20_2 (D)	20 × 20—over-use	2.467	+4.3
20x20_3 (D)	20 × 20—over-use	2.534	+7.1
20x20_5 (D)	20 × 20—over-use	2.677	+13.1

V: Klockner^®^; D: ZFX^®^/ZIMVIE^®^. Δ vs. Ref: relative change in maximum cross-sectional area with respect to the mean of the two unloaded reference screws of each system. All values exclude the two screwdriver-engagement sections. Klockner conditions: 10 × 30 N·cm (recommended), 20 × 30 N·cm (over-use), 10 × 40 N·cm (over-torque). ZFX/ZIMVIE conditions: 10 × 20 N·cm (recommended), 10 × 30 N·cm (over-torque), 20 × 20 N·cm (over-use).

## Data Availability

The raw data supporting the conclusions of this article will be made available by the authors on request.
